# Patient Selection for Deep Brain Stimulation for Pantothenate Kinase-Associated Neurodegeneration

**DOI:** 10.5334/tohm.929

**Published:** 2024-10-17

**Authors:** Jason L. Chan, Ashley E. Rawls, Joshua K. Wong, Penelope Hogarth, Justin D. Hilliard, Michael S. Okun

**Affiliations:** 1 Norman Fixel Institute for Neurological Diseases, University of Florida, Gainesville, Florida, USA; 2 Department of Neurology, University of Florida, Gainesville, Florida, USA; 3 Department of Molecular and Medical Genetics, Oregon Health & Science University, Portland, Oregon, USA; 4 Department of Neurosurgery, University of Florida, Gainesville, Florida, USA

**Keywords:** DYT-PANK2, globus pallidus internus, medication refractory dystonia, neurodegeneration with brain iron accumulation, neuromodulation

## Abstract

**Clinical Vignette:**

A 23-year-old woman with pantothenate kinase-associated neurodegeneration (PKAN) presented with medication-refractory generalized dystonia and an associated gait impairment.

**Clinical Dilemma:**

Bilateral globus pallidus internus (GPi) deep brain stimulation (DBS) can be an effective treatment for dystonia. However, outcomes for PKAN DBS have been variable and there are no standardized criteria for patient selection.

**Clinical Solution:**

Bilateral GPi DBS implantation resulted in improvement in dystonia and gait. The benefit has persisted over one year after implantation.

**Gap in Knowledge:**

PKAN is a rare neurodegenerative disorder and evidence supporting the use of PKAN DBS has been largely limited to case reports and case series. Consequently, there is a paucity of long-term data, especially on gait-related outcomes.

**Expert Commentary:**

The clinical characteristics of dystonia that respond to DBS tend to respond in PKAN. Clinicians counselling patients about the effects of DBS for PKAN should thoughtfully discuss gait and postural instability as important aspects to consider, especially as the disease will progress post-DBS.

## Clinical Vignette

A previously healthy, developmentally normal 23-year-old right-hand-dominant woman with pantothenate kinase-associated neurodegeneration (PKAN) presented for consideration of deep brain stimulation (DBS) candidacy. At 15 years of age, she developed opisthotonus when running and she would frequently toe walk. She had progressive handwriting changes which were accompanied by hand cramping and slurring of her speech. Her opisthotonic posturing and lower extremity dystonia became progressively sustained during walking. Gradually, she required a walker and subsequently, the use of a wheelchair. Her gait improved with skipping or walking backwards. Treatment with trihexyphenidyl, baclofen, and botulinum toxin injections improved her dystonia and enabled her to ambulate independently for short periods of time. Over the next several years, her speech, handwriting, and gait deteriorated. She gradually lost the ability to walk independently. She did not have other symptoms associated with PKAN, including visual or neuropsychiatric symptoms.

**Figure 1:**
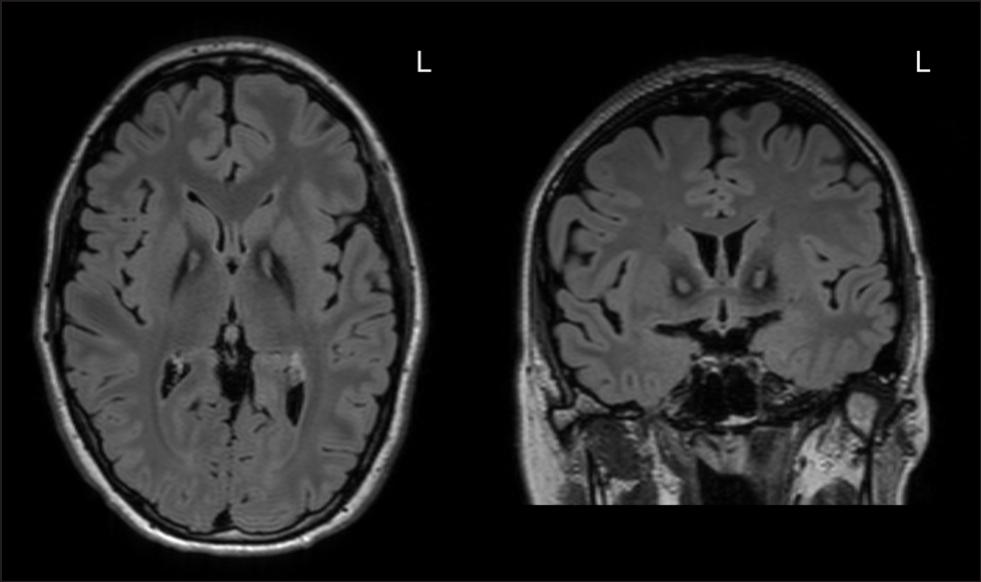
Axial (left) and coronal (right) T2-FLAIR brain MRI showed bilateral hypointensity of the globus pallidus with anterior medial hyperintensity, consistent with the “eye of the tiger” sign. L = left.

Her neurological examination revealed characteristics of adductor spasmodic dysphonia and generalized dystonia (Video 1). Her dystonia was most prominent when ambulating, which amplified retrocollis, opisthotonus, right upper extremity flexion, and bilateral plantar flexion. She had mild rigidity in the right upper extremity with activation maneuvers. Her brachioradialis, patellar, and Achilles reflexes were hyperreflexic. The remainder of the neurological examination, including a pull test, was unremarkable. Her brain magnetic resonance imaging (MRI) showed bilateral T2-fluid attenuated inversion recovery (FLAIR) hypointensities of the globus pallidus region with anterior medial hyperintensity, which was consistent with the “eye of the tiger” sign (Figure 1). Genetic testing revealed a missense variant (c.1255A>G, p.Ile419Val) on one allele of the pantothenate kinase 2 (*PANK2*) gene and deletion of exons 1–4 on the other allele.

**Video 1:** **Pre- and post-DBS examination.** Presurgical baseline examination at 23 years of age and postsurgical gait 15 months after bilateral GPi DBS implantation. Laryngeal dystonia was present with speech. Dystonia was most prominent when walking and impaired her gait. Retrocollis, opisthotonus, right upper extremity flexion, and bilateral plantar flexion were present. DBS improved her dystonia and gait.

## Clinical Dilemma

Bilateral globus pallidus internus (GPi) DBS can be an effective treatment for medication-refractory dystonia and has been associated with sustained benefit in dystonia severity, disability, and quality of life [1,2,3]. Outcomes can be influenced by the etiology and characteristics of dystonia. Some genetic dystonias, such as DYT-TOR1A, tend to be more responsive to DBS [4,5], whereas benefit for facial dystonia, speech, swallowing, and gait tend to be less predictable, especially at long-term follow-up [6]. Although outcomes for monogenetic dystonias are generally positive, benefit from DBS has been variable [7]. Dystonia resulting from neurodegeneration with brain iron accumulation (NBIA), and PKAN (DYT-PANK2) specifically, has been shown to improve following DBS [8,9]. However, the current evidence has been limited to case reports and case series, which are subject to publication bias and a lack of long-term data. Our case illustrates the challenges in predicting benefit from DBS for PKAN/NBIA, especially for gait.

## Clinical Solution

**Figure 2:**
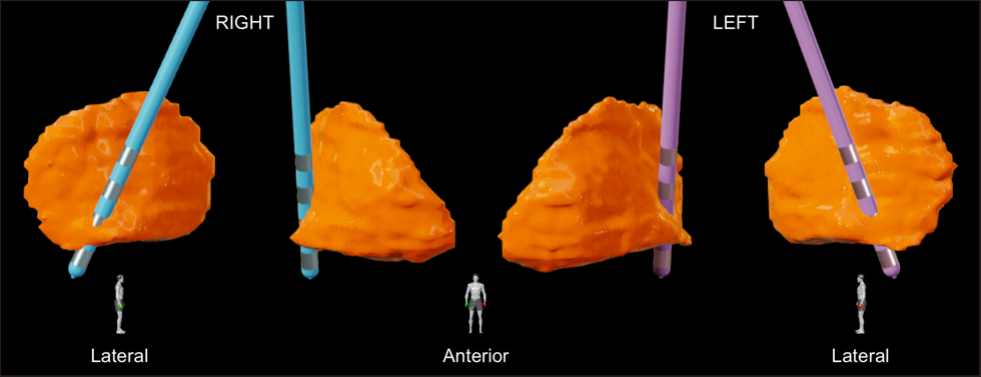
Lead localizations in the right (blue) and left (purple) globus pallidus internus (GPi) (orange), viewed from the lateral and anterior perspectives. Lead localization was performed using Brainlab Elements (Brainlab AG, Munich, Germany). Leads were implanted targeting the posteroventral GPi.

The patient was assessed by members of an interdisciplinary DBS team, including movement disorders neurology, neurosurgery, neuropsychology, psychiatry, physical therapy, occupational therapy, speech-language pathology, and social work. Following the interdisciplinary assessment, it was determined that she was a reasonable candidate for bilateral GPi DBS. She underwent bilateral GPi DBS surgery without surgical complication. Post-operative head CT and lead localization confirmed appropriate lead placement (Figure 2), which was also supported by thresholds for side effects during device programming. One month following initial DBS programming (10- C+ with 2.5 mA, pulse width 90 µs, and frequency 135 for the right GPi, and 2- C+ with 2.5 mA, pulse width 90 µs, and frequency 135 Hz for the left GPi), she reported minimal to no dystonia in her upper extremities, minimal truncal dystonia, and improved lower extremity dystonia. She reported a 75% improvement in her gait and was able to independently walk over a mile per day. She has not had further in-person follow-up at our center, as she lives 1,000 miles away. Through personal communication, she reported a sustained response to DBS over a one-year period (Video 1).

## Gap in Knowledge

NBIA is a group of rare neurodegenerative disorders characterized by iron accumulation in the globus pallidus and substantia nigra. PKAN represents up to 50% of NBIA cases with an estimated prevalence of 1 in 1,000,000 and is caused by biallelic pathogenic variants in the *PANK2* gene [10,11]. “Classic PKAN” presents in early childhood, with a mean age of onset of 3 years, and is characterized by dystonia and spasticity, which contribute to gait impairment and dysarthria. Pigmentary retinopathy and developmental delay are common. “Atypical PKAN” has a later onset, commonly after 10 years of age, and is characterized by speech impairment, dystonia, spasticity, and parkinsonism. Neuropsychiatric symptoms, including impulsivity and obsessive-compulsive behaviours, commonly occur. Disease progression is faster in classic PKAN compared to atypical PKAN and the development of postural instability contributes to falling.

Progressive dystonia is frequently the most disabling motor symptom in PKAN and dystonia treatment is one of the primary goals of PKAN management [12]. The general principles for patient selection and management in DBS for dystonia can be applied to PKAN. GPi DBS may be more effective for dystonia of the limbs, neck, and trunk compared to dystonia involving laryngeal or oromandibuar musculature [13]. Outcomes are less favourable when dystonia involves tonic posturing [14] and DBS is less effective or ineffective when musculoskeletal deformities such as fixed joint contractures and scoliosis are present [15,16]. Accordingly, the body regions affected by dystonia, range of motion, and presence of deformities are crucial factors for pre-DBS PKAN assessments.

Published cases of PKAN DBS provide some insight on patient selection. The largest meta-analysis to date included 99 patients and reported that the type of PKAN, classic or atypical, was predictive of outcome after one year of GPi DBS [8]. In this study, classic PKAN was defined as symptom onset or DBS surgery before 10 years of age and atypical PKAN was defined as symptom onset at 14 years of age or older. Genetic confirmation of diagnosis was not available in all cases and thus other NBIA etiologies may have been included. Atypical PKAN was associated with a 45% decrease (improvement) in the Burke-Fahn-Marsden Dystonia Rating Scale (BFMDRS) motor score compared to only a 16% decrease (improvement) in classic PKAN. BFMDRS disability scores improved in atypical PKAN but were unchanged in classic PKAN. Better outcomes in the atypical PKAN group were hypothesized to be related to higher levels of baseline motor skills, perhaps as a result of later disease onset or slower disease progression.

Outcomes in PKAN DBS differ when compared to primary dystonia, including DYT-TOR1A, when considering disease duration and dystonia severity. Longer disease duration [15,17] and more severe symptoms [18] in primary dystonia, as well as the presence of fixed musculoskeletal deformities, have been associated with reduced DBS-related benefit. In contrast, disease duration may not be as predictive of outcome in PKAN DBS [8] and more severe pre-operative dystonia has been associated with greater benefit [8,9]. Rapid dystonia progression in PKAN may result in severe dystonia with less musculoskeletal deformities, and this may explain its responsiveness to DBS.

There is a paucity of long-term outcome data for PKAN DBS. Nine reported cases of PKAN DBS followed for a minimum of four years showed that 8 of 9 cases maintained improvement in dystonia when compared to their preoperative baseline [19,20,21,22]. All cases of PKAN with long-term follow-up experienced decrements in motor outcome. PKAN disease and dystonia progression have been hypothesized to underlie this worsening [23]. DBS stimulation parameters can be adjusted over time to potentially address worsening symptoms. In 2 cases that reported parameter adjustments, the degree of additional DBS benefit may have been limited by worsening spasticity [21] or fixed deformities [19]. The improvement in atypical cases, which present later in life and tend to progress more slowly, suggests that slower rates of progression may be a positive predictor.

Optimal lead placement, the choice of lead contact(s) for stimulation, and the selection of stimulation parameters in PKAN DBS should be guided by experience with DBS for other forms of dystonia. Suboptimal lead placement is associated with worse outcomes [16]. Leads placed in the sensorimotor region of the GPi have been effective for dystonia [24,25] and we believe that this data-driven observation is likely true for dystonia in PKAN. Optimal stimulation parameters may be challenging to identify due to the absence of immediate symptomatic benefit following device programming and maximal dystonia benefit may take months or longer to achieve. Interestingly, our case had substantial dystonia improvement within one month of activating DBS. Several other cases of PKAN DBS have reported similarly rapid improvement within 2–6 weeks of stimulation [19,20,26]. Further investigation will be required to determine whether this is a generalizable phenomenon in PKAN DBS.

Subthalamic nucleus (STN) DBS has also been shown to be effective for dystonia [27,28], and STN DBS has been beneficial for both classic and atypical PKAN [29,30]. STN DBS may have a faster onset of benefit and greater benefit for limb dystonia compared to GPi DBS [28]. Our case had a relatively rapid response to stimulation that may be comparable to STN DBS. Overall, the literature on STN DBS for dystonia has been limited and the GPi has been the most established target for medication-refractory dystonia.

## Expert Commentary

DBS is a symptomatic treatment for dystonia and dystonia phenomenology commonly predicts DBS outcome regardless of etiology. The body regions affected by dystonia, range of motion, and presence of deformities are factors important for patient selection. Greater improvement in atypical PKAN compared to classic PKAN suggests that the level of learned motor skills is an additional consideration. Clinicians approaching discussions on potential DBS outcomes for gait should thoughtfully consider the impact of postural instability. DBS treats dystonia, which may contribute to gait impairment, but does not improve postural instability. The “pull test” is an important bedside maneuver that assesses a patient’s degree of postural instability and should be documented pre-operatively and performed at regular intervals throughout the lifetime of an individual with PKAN. An abnormal pull test may indicate an increased risk for falls, fractures, and related comorbidities. Our patient had a relatively preserved preoperative postural response and her gait improved as a result of a reduction of dystonia from DBS. Since PKAN will progress over time, serial examinations of postural instability will be required along with other proactive strategies to maximize fall prevention.
